# Measuring the impact of chronic low back pain on everyday functioning: content validity of the Roland Morris disability questionnaire

**DOI:** 10.1186/s41687-020-00234-5

**Published:** 2020-08-28

**Authors:** Claire Burbridge, Jason A. Randall, Lucy Abraham, Elizabeth Nicole Bush

**Affiliations:** 1 Clinical Outcomes Solutions, Unit 68 Basepoint, Shearway Business Park, Shearway Road, Folkestone, Kent, CT19 4RH UK; 2 Pfizer Ltd, Tadworth, UK; 3 Eli Lilly and Company, Indianapolis, USA

**Keywords:** Content validity, Concept elicitation, Cognitive debriefing, Chronic low back pain, PRO development, Roland Morris Disability Questionnaire (RMDQ)

## Abstract

**Background:**

Robust outcome measures are needed to assess and monitor the impact of chronic low back pain (CLBP) on physical functioning. The Roland Morris Disability Questionnaire (RMDQ) is a well-established measure designed to capture the impacts of back pain on everyday functioning, with a particular emphasis on physical functioning. It has documented evaluation of psychometric properties. However, there is no documented qualitative evidence to confirm the content validity of the tool, nor have changes made for electronic administration been debriefed in participants with CLBP.

**Methods:**

In-depth, semi-structured, concept elicitation and cognitive debriefing interviews were conducted with 23 US participants with confirmed CLBP. Interviews allowed participants to describe the impact of CLBP on their day-to-day functioning and discuss comprehension and suitability of the RMDQ. Interviews were transcribed verbatim and analyzed using thematic analysis.

**Results:**

Concept elicitation and cognitive debriefing revealed the substantial burden associated with CLBP, highlighting 15 key areas of functional impact. These were grouped into overarching themes of mobility (walking, stairs, sitting/standing, bending/kneeling, lifting, lying down), activities (chores/housework, dressing, washing, driving, work) and other (relationships/socializing, mood, sleep, appetite), which are consistent with those evaluated within the RMDQ.

All participants found the RMDQ to be relevant with most reporting that the instructions, recall period, and response options were suitable. A few suggested minor changes, however, none were consistent or necessary to support content validity. Updates to the measure for electronic administration and to clarify the response options were well received.

**Conclusion:**

The qualitative data from individuals with CLBP confirmed that the RMDQ has content validity and, alongside documented psychometric evidence, supports the use of the RMDQ as a reliable and valid tool to assess the impact of CLBP on physical functioning.

## Background

Chronic low back pain (CLBP) is a common debilitating condition that affects many people worldwide [1]. A 2015 systematic review of studies evaluating CLBP in adults reported that all studies showed an increasing prevalence of CLBP with age; the lowest prevalence rates were in the younger age group (aged 20 to 30 years) and rates increased to peak in those 50 to 60 years of age [1]. CLBP has a substantial burden on both the individual and society, with high levels of disability and physical function impairment, sick leave and work loss, greater health care utilization and treatment costs, and an increased risk of coronary events and other comorbidities all commonly reported [2–5].

Robust outcome measures are needed to assess and monitor the impact of CLBP on physical functioning. The Roland Morris Disability Questionnaire (RMDQ) [6] is a well-established measure, with documented evaluation of psychometric properties, which is widely used in clinical trials [7–14]. The RMDQ was developed to capture the everyday functional impact of CLBP. Although it does include some broader concepts than might traditionally fall within a strict definition of physical functioning, due to the nature of the condition, it is primarily focussed on physical functioning (mobility, ability to carry out activities of daily living) [6, 15]. The RMDQ is thus acknowledged and used as a measure of physical function in CLBP [16]. It has been shown to have test-retest reliability (*intra-class correlation coefficient [*ICC] > 0.70) [6, 17–20] and internal consistency reliability (Cronbach’s alpha > 0.80) [20–22]. In research demonstrating construct validity, strongest correlations have been reported with other measures of self-reported disability and health-related quality of life (the Oswestry Disability Index: 0.79–0.80^23^ and SF-36: 0.60–0.85) [22] and weakest correlations are reported with objective tests of physical function [23, 24] such as fingertip to floor (r = 0.27) [23], straight leg raise (r = 0.44) [23], and 15 m walk test (r = 0.37) [24]. These correlations reflect the pattern of relationships that would be expected for a measure of self-reported physical function in CLBP. There is strong evidence of ability to detect change on the RMDQ from its use in many CLBP clinical trials with several compounds, including duloxetine, amoxicillin/clavulanic acid, carisoprodol, diclofenac, etoricoxib, glucosamine, hydromorphone, rofecoxib, and tanezumab [7–14]. The Initiative on Methods, Measurement, and Pain Assessment in Clinical Trials (IMMPACT) guidelines include the RMDQ as an example of a disease-specific measure that has been developed to evaluate physical function in CLBP [16].

The measure has also been successfully used in a United States (US) label claim for carisoprodol (Soma®), indicated for the relief of discomfort associated with acute, painful musculoskeletal conditions in adults, as a secondary outcome measure to support improvement in function. However, this label claim approval was given in 2009, before publication of the final Food and Drug Administration (FDA) patient-reported outcome (PRO) guidance [25].

While the RMDQ is a commonly used measure and performs well as a measure of physical functioning in CLBP, a literature review and gap analysis undertaken as part of this project identified a lack of documented content validity evidence. Although the common use of the RMDQ may suggest acceptance within the clinical community of content validity, it is necessary for this to be documented and shown qualitatively through direct feedback from patients to meet current standards of good practice [25–27]. No qualitative studies exploring the content validity of the RMDQ in participants with CLBP could be identified.

Content validity is a crucial property of a measure, which shows that all concepts of interest from the patient perspective have been adequately captured in the measure. Establishing content validity is an essential component of demonstrating that a PRO measures the intended concept and is fit for purpose, and this requires obtaining insights and feedback directly from patients through qualitative research to ensure that their voice is appropriately captured. The FDA PRO guidance makes clear that any additional validation builds upon this foundation [25].

The original version of the RMDQ asked participants to mark only those items that are relevant to them and leave those that are not blank. This format created an issue whereby it was not possible to know if “no response” meant that the statement was not applicable to the respondent that day, or if the item had been missed and thus represented missing data. To combat this, the study team made minor modifications to the response options of the RMDQ during migration to electronic format. The dichotomous response option has been made explicit, rather than being implied. In the modified version, respondents are asked to indicate a “yes” or “no” response to each item. In addition, the authors updated the mode of administration from the original paper-based format, to an electronic version for administration via a tablet device. The usability and feasibility of this electronic mode of administration were evaluated in a separate study [28]. Since there is a lack of documented qualitative evidence to support the content validity of the RMDQ or to support the minor modification to the response options, current regulatory acceptance cannot be assumed. Therefore, it is also necessary to debrief the updated version of the RMDQ in qualitative interviews.

The purpose of the current research is therefore to explore the content validity of the RMDQ; to qualitatively explore the patient experience of CLBP to understand what aspects of everyday functioning are most impacted by CLBP; and to explore the relevance, comprehension and patient understanding of instructions, recall period, and response options of the RMDQ.

## Methods

### Study design and participants

In-depth, semi-structured, face-to-face interviews were conducted. The study was designed and conducted in line with established research practices, including the guidelines provided by the ISPOR taskforce [29] and the Declaration of Helsinki and US 21 Code of Federal Regulations [30].

Inclusion criteria included a clinician confirmed diagnosis of CLBP for ≥3 months without radiation to the posterior thigh. Individuals with any comorbidities or recent surgery or trauma that could affect pain perception or participation in an interview study were excluded; this was on the basis of the recruiting clinician opinion, however examples of other conditions and surgeries to be excluded were given to guide this (Conditions: osteoporosis, rheumatoid arthritis, fibromyalgia, spinal stenosis, CLBP due to visceral disorder, seronegative spondyloarthropathy and neurogenic claudication; Surgeries: discectomy, nerve ablation in the back, kyphoplasty and nucleoplasty). Participants were identified using purposive sampling by a recruitment agency from three locations within the US: Baltimore, Maryland; St Louis, Missouri; and Los Angeles, California. This was to maximize geographic diversity and to allow for a broad spectrum of participants to be recruited for the study. Following identification, participants were invited to attend the clinic to discuss the study and to provide written informed consent. All participants who signed the consent form were enrolled and no participants withdrew from the study. The eligibility of consented participants was confirmed by their clinician who completed a Case Report Form detailing eligibility and medical history for all interested potential participants after informed consent was obtained.

Participants also completed a demographic health information form prior to conducting the interview.

### Interviews

The details of those providing informed consent were passed to the researchers and participants were contacted to arrange a mutually convenient time for an interview. All interviews were completed by an experienced qualitative researcher. Interviews were undertaken in a hotel meeting room local to the participants, arranged so that all interviews from a particular site could be conducted within a two-day period. Prior to the interview, the interviewer worked on developing a rapport with the participant, sharing information about who they are, where they work, and the purpose of the interview. All interviews were audio-recorded and then transcribed verbatim and de-identified.

The interviews involved concept elicitation (CE) and cognitive debriefing (CD) utilizing a semi-structured interview guide. Following a general introduction to start, the first part of the interview was the concept elicitation phase in which the participant was asked about their CLBP and its impact on his or her everyday functioning. This included questions such as “Can you describe the symptoms of your back pain, for example, how do the symptoms feel?”, “How often do you experience symptoms?”, and “How does having back pain impact you and your life?” The second phase of the interview was the cognitive debrief of the RMDQ. Participants were asked to complete the modified version of the RMDQ using the updated yes/no response options. Screenshots of the RMDQ from the electronic device were presented to the participant for pen and paper completion. This enabled participants to see how the measure would be presented in electronic format, so allowing any changes to formatting or layout to be seen. After completing the measure, participants were asked for feedback on the instructions, items, recall period, and response options, specifically whether they were understandable, relevant, and comprehensive. The interviewer then asked the participants to review the overall content of the measure to determine whether it covered all relevant functional impacts that are important to them.

### Analytical approach

De-identified transcripts were uploaded to ATLAS.ti version 7.0. Coding was undertaken and the coder (second author) met regularly with the interviewer to discuss codes, as well as any non-verbal cues and to make sure any interviewer notes were incorporated. The coding was also reviewed by the project lead (first author), who reviewed the coding for a selection of transcripts and engaged in coding discussions to ensure codes had been applied consistently and accurately and that any coding discrepancies were reconciled via consensus within the research team.

The concept elicitation data were analyzed using inductive thematic analysis [31]. The cognitive debrief section of the interviews was coded to focus on participant input pertaining to the main research questions, including relevance, comprehension, and any rewording suggestions.

Saturation analysis was undertaken, using both spontaneous and probed responses, by dividing the sample into three equal groups, based on the chronological order in which participants were interviewed. Saturation was considered met when no new topics were discussed in the final group of participants, thus it was deemed that no more interviews were necessary. Saturation analysis is only undertaken on concepts identified through concept elicitation, as it is appropriate only when there is an open-ended participant-led discussion during which concepts are being explored (ie, CE interviews).

### Ethics

An Ethical Review Board (Copernicus Group Independent Review Board, US; protocol #A4091073) approved the study and study documents. Participants received a small stipend of US $125 for their participation.

## Results

A total of 23 individuals (11 males [47.8%], 12 females [52.2%]) with a clinician confirmed diagnosis of CLBP for ≥3 months without radiation to the posterior thigh were interviewed; all based in the US (7 from Baltimore, 7 from St Louis, and 9 from Los Angeles). Participants were aged between 23 and 73 years (mean 53.9 years) and reported mild to very severe pain (3–10 on a 0–10 numeric rating scale; mean 6.5). The mean time since diagnosis was 7.86 years (ranging from 0.4–30.09 years), none had undergone surgery for CLBP, and 22 were currently receiving treatment (one participant did not answer this question on the demographic health information form). Demographic and descriptive data for the participants are presented in Table 1.

**Table 1:** Additional demographic and health information of participants (*N* = 23)

		N	%
Race	White/Caucasian	16	69.6
	Black/African America	5	21.7
	American Indian/Alaska Native	1	4.3
	Other (stated as minority)	1	4.3
Ethnicity	Hispanic/Latino	3	13.0
	Non-Hispanic/Latino	20	87.0
Education	Did not complete high school	4	17.4
	High school diploma (or GED)	5	21.7
	Some college or certificate program	8	34.8
	College or university degree (2- or 4-year)	4	17.4
	Graduate degree	1	4.3
	Other	1	4.3
Employment	Employed full-time (= 40 h per week)	13	56.5
	Employed part-time (< 40 h per week)	1	4.3
	Homemaker	0	N/A
	Student	0	N/A
	Retired	7	30.4
	Unemployed	0	N/A
	Other (both participants self-employed)*	2	8.7
If retired, is this due to CLBP? (*n* = 7)	Yes	5	71.4
	No	2	28.6

*Being ‘self-employed’ was not differentiated in the demographic form as a separate option to ‘employed’ however, two participants indicated this within the ‘other’ category

**Fig. 1:**
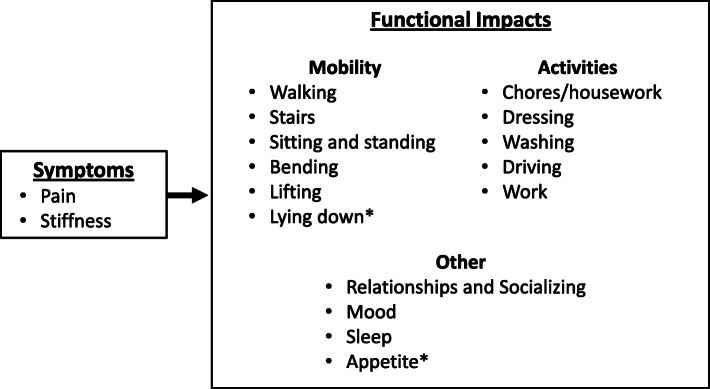
Conceptual Model of CLBP from the Participant Perspective. CLBP – chronic low back pain. * These themes were identified as important during the cognitive debrief phase of the interview and were not spontaneously mentioned during concept elicitation

Overall, analysis of the qualitative data revealed a substantial burden associated with CLBP. Throughout the interviews, individuals with CLBP discussed a range of functional impacts, particularly focusing on physical functioning, all reported as salient to the individual with CLBP. A conceptual model was developed to represent this (Fig. 1), using language that was used by the participants during the interviews. For ease of illustration and interpretation, these 15 areas were grouped into overarching themes of mobility (walking, stairs, sitting and standing, bending and kneeling, lifting, lying down), activities (chores/housework, dressing, washing, driving, work), and other (relationships and socializing, mood, sleep, appetite).

The majority (13 of the 15) physical function impact areas identified as being important to individuals with CLBP were spontaneously discussed in the initial concept elicitation section of the interviews. A further two key areas of impact (lying down and appetite) were first raised during the cognitive debriefing of the RMDQ. Although these concepts were not discussed in depth as part of the CE section, when discussed during CD these were clearly identified by the majority of participants as being highly relevant (23/23 and 21/23 for the lying down and appetite items, respectively), the former being a common way of relieving pain or resting due to back pain, and the latter being affected by severe back pain. Thus, these were felt to be important concepts to capture and therefore these were pulled out as part of the theme development. Thus, all participant-derived qualitative data was utilized, from both sections of the interview, to inform a complete picture of the participant experience of CLBP as presented in the conceptual model.

Saturation analysis was undertaken on the 13 concepts identified during concept elicitation, with no new concepts being identified in the last round of interviews. Therefore, saturation was met.

Table 2 presents each theme from the model with quotes from the interviews that illustrate the importance of the theme to the individuals. As can be seen from the number of participants reporting each theme, there was substantial commonality between individuals in the key areas of function impacted by CLBP.

**Table 2:** Key qualitative themes

Theme		Example quote	Number of participants
Walking	Walking more slowly	“No. I walk, [I just] I don’t walk as fast” P01–006	21/23
	Can only walk short distances	“Walking, I can’t walk for long periods of time even though I try. I … that’s what one of my exercises. I try to push myself” P01–002	
Stairs	Using a handrail on stairs/steps	“So I’m just real careful when I walk, and I grab handrails when I go down stairs.” P02–007	10/23
	Go upstairs more slowly	“I have a hard time going up and down steps … I have to hold the railing and I have to do one step up. One step up. One step [at a time moving slowly]-” P03–004	
Sitting and standing*	Unable to sit or stand for long periods of time	“Yeah, I can’t stand ... I mean, I can’t stand too much, I can’t sit down too much.” P01–007	Sitting 23/23; 8/23 also discussed problems with standing
	Changing position to get comfortable	“Sitting for a long time and standing up, uh, takes a while to, you know, get to where I can, I’m comfortable moving forward I need to get up … Get the, you know, the soreness out. Because if I, if I sit for a long period, it’s really difficult” P01–001	
	Holding something to get in/out of a chair	“at certain times, when I’m either getting up out of a chair, or sitting in a chair, and that it feels like my back has gone out, then I have to really grab on to something.” P03–007	
	Difficulty getting out of a chair	“I mean sometimes it’s just getting up out of a chair, or sometimes it’s sitting in a chair [which cause pain], you know what I mean?” P03–007	
	Sitting down more	“And sometimes if it’s, you know, a 7 or 8 [pain] day, then I’ll sit in the recliner with the heating pad.” P02–003	
Bending and kneeling	Pain while bending	“it’s just pain like on my lower back. Like, say if I bend over” P01–003	12/23
	Pain while kneeling	“Um, probably like taking stuff out of the washer, to bend down to put it into the dryer … or clean the litter box, when you’re, you know, when you’re ... kneeling and scooping [are more difficult because of the CLBP]” P02–003	
	Avoiding bending/kneeling or using assistance	“[my wife] Ties my shoes for me. Uh, like, uh, bending over is, uh, hard” P01–005	
Lifting	Pain while lifting	Lifting. I, I occasionally do land-scaping at our house and lifting bags of, uh, mulch, bags of dirt, um, doing those a lot definitely brings on the back pain” P02–004	15/23
	Avoiding lifting	“Uh, I’ll be honest with you. I don’t do nothing heavy. Anything that’s probably 50 pounds or more, I really stay away from that kind of stuff” P01–005	
Lying down	Lying down was not spontaneously reported in the concept elicitation section of the interviews, it was highlighted as important and relevant to 23/23 participants during the cognitive debriefing of the RMDQ, and as such was pulled into the conceptual model.		
Chores/ housework	Avoiding/not doing as many jobs around the home	“Sometimes, just when I have severe pain or when I feel an onset coming on......I feel like I can’t function correctly. So, I sometimes just decide not walk my dog, clean the house or wash my car.” P01–008	23/23
	Being slower/not getting as much done	“You know, so instead of knocking half my list on a weekend I might, just get one project done. Just take my time with it”. P02–001	
	Getting other people to do jobs	“I hire young men with strong backs to do jobs around the home [as I can’t do it now].” P02–007	
Dressing	Trouble putting on socks, shoes and trousers	“I’ve not been able to put on my socks and shoes and struggle mightily with my pants” P02–004	19/23
	Needing help to get dressed	“my girlfriend puts my socks on for me and my shoes … So she [girlfriend] does the pants, you know” P01–005	
	Getting dressed more slowly	“You give yourself a little bit more time to get dressed once in a while or a little bit more time to go somewhere” P02–008	
Washing	Difficulties with washing	“Um, well, you know, in the shower, again, I’m very conscious about all movements, um luckily we have grab bars and things in there. I utilize those. Um, minimize twisting, uh bending, you know all that sort of thing” P02–001	11/23
Driving	Getting in and out of cars	“[getting] Out of, out of a car, certain cars … The lower the chair or the, whatever you’re sitting on … the harder” P02–008	9/23
	Remaining in the same position long periods of time	“once that happens [Pain from CLBP], I am not able to go out to work. Um, just because I, I’ll normally be a long time of period in the car [and I can’t sit for that period of time].” P01–008	
Work^#^	Not able to work or do same job/taking a break at work	“Um, to the point at work where I actually have to stop working for that little bit of time, to try and ease the pain in my back” P03–007	12/23
		“Uh-huh (affirmative), because now, yeah, not working because I always have a pain, so I do the best I can” P01–007	
Relationships and socializing	Missing events/Staying at home	“I mean, family picnics, … we missed Christmas Eve with the family. [we have missed] All kinds of family functions over the years. You know, we’ve been married for, uh, 21 years so there’s been lots of things over the years [we have missed because of the CLBP].” P02–004	17/23
	Relationships with children and family	“my kids I could never do anything with them. I couldn’t play football, or baseball. I tried to coach little league with my son and I couldn’t do it. It was too much pain. I tried to play softball myself and I couldn’t do it. I had to, I had to quit because I couldn’t run”. P03–006	
	Sexual activities	“Um, I haven’t had sex for over, maybe 15 years. [because of my CLBP]” P03–004	
	Support system	”I have very strong support system, very strong, um, family ties” P01–002	
Mood	Moody, irritable and short tempered	“Oh, yeah. I think, uh, I feel like I’m more agitated easily. I have mood swings, constantly” P01–008	20/23
	Depression and anxiety	“Um, I, I get depressed because of, thinking about things that I used to do, that I can’t do, and there’s a whole lot of other things” P03–004	
Sleep	Hard to fall asleep	“Oh, yes. Um, I find it hard to fall asleep sometime” P01–008	20/23
	Waking up	“It’s, um, say, say when I’m lying in bed sleeping at night … I, I wake up and, and, and I got to ... and I can’t move. I got to get up. I’ve got to sit up and I’ve got to sit there for a minute because it hurts so bad” P03–006	
	Can’t get comfortable	“like I’m tossing and turning. I try to do the lying on the side, put the pillow between my legs for my spine and, uh, I tried the pillows up, raising my feet up. You know, I, I did different things” P01–005	
Appetite	Appetite was not spontaneously reported in the concept elicitation section of the interviews, it was highlighted as important and relevant to 21/23 participants during the cognitive debriefing of the RMDQ, and as such was pulled into the conceptual model.		

*Difficulties with standing were only discussed with difficulties with sitting and as such these topics were combined as an example of similar limitations

^#^Not all participants engaged in paid work

As in traditional PRO development, concepts identified from the interviews were reviewed to identify areas of physical function impairment that should be included in a comprehensive measure for CLBP. Items from the RMDQ were then reviewed to determine the retrofit against identified concepts. This is presented in Table 3. In some instances, there is a direct relationship between the themes identified in the interviews and a single RMDQ item. Reflecting the multi-dimensional nature of CLBP, many themes identified within the interviews represent multiple examples of activities that are impacted by the pain and associated mobility issues or physical function limitations. In these cases, several RMDQ items can be considered as capturing the broad impact of these topics being discussed in the interviews. Table 3 clearly shows that the RMDQ items capture all impacts on everyday physical function that were identified as being important to participants.

**Table 3:** Mapping the themes identified in coding to the RMDQ items

RMDQ - item number and summary	Overarching concept	Theme	Subcode	Note
1 – I stay at home most of the time because of my back	Other	Relationships and Socializing	Missing events/staying at home	Participants reported missing events due to the functional limitations they experience from their CLBP. This is a functional consequence of the physical limitations experienced. Those who engaged in paid work also reported missed work days due to pain.
	Activities	Work*	Not able to work or do same job/taking a break at work	
2 – I change position frequently	Mobility	Sitting and standing	Changing position to get comfortable	Participants reported having to change positions regularly to reduce their CLBP. This created problems with various activities that require sitting or standing in one position for a long period of time. Thus, this a physical functioning limitation due to CLBP. Driving and certain types of work are examples of specific activities discussed in relation to this.
	Activities	Driving*	Remaining in the same position for long periods of time	
	Activities	Work*	Not able to work or do same job/taking a break at work	
3 – I walk more slowly	Mobility	Walking	Walking more slowly	Participants reported having to walk more slowly because of the pain and need for smaller steps/avoiding tripping. This is a physical functioning limitation due to CLBP.
4 – Not doing jobs around home	Mobility	Lifting	Pain while lifting Avoiding lifting	Participants reported that they cannot do jobs around the home like they used to because of their pain and other physical limitations due to their CLBP. Participants talked about engaging in a range of chores such as mopping the floor or taking the trash out, as well as the action needed to perform jobs such as lifting things, Thus, this is a physical functioning limitation due to CLBP.
	Activities	Chores/housework	Avoiding/not doing as many jobs around the home Being slower/not getting as much done	
5 – I use a handrail to get upstairs	Mobility	Stairs	Using a handrail on stairs/steps	Participants reported having to use a handrail when using stairs/steps because of pain and limited mobility due to CLBP. Thus, this is a physical functioning limitation due to CLBP.
6 – I lie down to rest	Mobility	Lying down	–	Participants identified this as important during the CD discussion, although this was not spontaneously discussed during CE. Participants reported lying down to rest because of their pain, and although not directly stated it was implied that this impacts upon functional ability as time spent lying down limits ability to take part fully in other daily activities. Work was an example of a specific activity impacted by the need to take a break, lie down and rest by those who engaged in paid work.
	Activities	Work*	Not able to work or do same job/taking a break at work	
7 – Hold on to get out of an easy chair	Mobility	Sitting and standing	Holding something to get in/out of chair	Participants reported needing to hold something to get out of a chair due to pain and limited mobility. This is a physical functioning due to CLBP.
8 – I try and get other people to do things for me	Mobility	Bending and kneeling	Pain while bending Pain while kneeling Avoiding bending/kneeling	Participants reported that their inability to do all their usual jobs, for example, because of being unable to lift things, having pain while bending and kneeling, meant that they would now get other people to do things for them. This is an everyday functional consequence of the physical limitations experienced. Washing (self) and housework are examples of specific activities discussed in relation to this, in addition this also came up for some individuals in relation to tasks that needed to be done whilst at work.
	Mobility	Lifting	Pain while lifting Avoiding lifting	
	Activities	Chores/housework	Getting other people to do jobs	
	Activities	Washing*	Difficulties with washing	
	Activities	Work*	Not able to work or do same job/taking a break at work	
9 – I get dressed more slowly	Activities	Dressing	Getting dressed more slowly	Participants reported they took longer to get dressed due to pain and limited mobility. This is a physical functioning limitation caused by CLBP.
10 – I only stand for short periods	Mobility	Sitting and standing	Unable to stand for long periods of time	Participants reported that they could not stand for long periods because of their pain. This is a physical functioning limitation due to CLBP.
11 – I try not to bend or kneel	Mobility	Bending and kneeling	Pain while bending Pain while kneeling Avoiding bending/kneeling	Participants reported that they could not bend or kneel because of their pain and so avoided doing this. This is a physical functioning limitation due to CLBP. Washing (self) and getting in/out of a car are examples of specific activities discussed in relation to this, as well as work for those who usually did work involving this type of action.
	Activities	Washing*	Difficulties with washing	
	Activities	Driving*	Getting in and out of cars	
	Activities	Work*	Not able to work or do same job/taking a break at work	
12 – I find it difficult to get out of a chair	Mobility	Sitting and standing	Difficulty getting out of a chair	Participants reported difficulty with getting out of a chair due to pain and mobility limitations caused by their CLBP. This is a physical functioning limitation of CLBP.
13 – Back is painful most of the time	Pain	Pain	Overall pain Background pain Extreme pain	Participants reported that they experienced pain, with some reporting a background pain that was present most of the time as a key symptom of the condition leading to the physical functioning limitations described.
14 – Turn over in bed	Other	Sleep	Cannot get comfortable	Participants reported that because of their pain, they struggled to get comfortable in bed and would toss and turn a lot. This created problems with sleep and is a physical functioning limitation as sleep is a daily function.
15 – Appetite is not very good	Other	Appetite	–	Participants identified this as important during the CD, although this was not spontaneously discussed during CE. Participants reported that when they were in pain they could lose their appetite. This is an example of a consequence of pain which could have everyday impact.
16 - Putting on socks (or stockings)	Activities	Dressing	Trouble putting on socks, shoes, and trousers	Participants reported that they had trouble putting on socks, shoes, and trousers because of pain and mobility limitations. This is a physical functioning limitation due to CLBP.
17 – Walk short distances	Mobility	Walking	Can only walk short distances	Participants reported they now can only walk short distances because of pain and mobility limitations. This is a physical functioning limitation due to CLBP.
18 – Sleep less well	Other	Sleep	Hard to fall asleep Waking up	Participants reported that because of their pain they found it hard to fall asleep and would often wake up during the night. This created problems with sleep and is a physical functioning limitation since sleep is a daily function.
19 – I get dressed with help	Mobility	Bending and kneeling	Pain while bending Pain while kneeling Avoiding bending/kneeling	Participants reported that they require help when getting dressed because of pain and mobility limitations. This is an everyday functional consequence of the physical functional limitations experienced. Bending and kneeling were discussed as one of the direct reasons for needing help (eg, cannot reach feet, pick items off the floor).
	Activities	Dressing	Needing help to get dressed	
20 – I sit down most of the day	Mobility	Sitting and standing	Sitting down more	Participants reported that they would often sit in a chair more to rest because of their pain. This is a functional limitation due to CLBP. Some participants also discussed how the need for rest could impact their work.
	Activities	Work*	Not able to work or do same job/taking a break at work	
21 – I avoid heavy jobs	Mobility	Lifting	Pain while lifting Avoiding lifting	Participants reported that they would avoid heavy jobs around the home (or for some also at work) because of pain and mobility limitations. Participants discussed that they would avoid undertaking jobs or activities that involved a lot of heavy lifting or movement such as lifting heavy objects, moving plant pots, heavy boxes, moving furniture, etc. This is a physical functioning limitation due to CLBP.
	Activities	Chores/housework	Avoiding/not doing as many jobs around the home	
	Activities	Work*	Not able to work or do same job/taking a break at work	
22 – Irritable and bad tempered with people	Others	Mood	Moody, irritable and short-tempered	Participants indicated that because of their pain and physical limitations they would become moodier and more irritable then they were before. This is an emotional consequence of the symptoms and functional limitations experienced.
23 – I go upstairs more slowly	Mobility	Stairs	Go upstairs more slowly	Participants reported that they move upstairs slower than they used to because of pain and mobility limitations. This is a physical functioning limitation due to CLBP.
24 – I stay in bed most of the time	Mobility	Lying down	–	Participants identified this as important during the CD, although this was not spontaneously discussed during CE. Participants reported lying down to rest because of their pain and staying in bed when it was at its worst and although not directly stated it was implied that this impacts upon functional ability as time spent staying in bed limits the ability to take part fully in other daily activities.

*CD* cognitive debriefing, *CE* concept elicitation, *CLBP* chronic lower back pain

*Washing, driving and work were identified in the conceptual model but not explicitly captured within the RMDQ as distinct items. These concepts were examples of activities impacted by the functional limitations experienced, and thus relate to a number of items as detailed in the table

Although three of the concepts (washing, driving, and work) identified in the conceptual model are not explicitly captured in the RMDQ, it is felt that these are encapsulated within other items or are activities that could be better captured using other more specific measures. For example, work could be impacted by any of the functional limitations experienced in CLBP, depending upon the nature of the job. Difficulties with washing and driving, upon discussion, were both due to functional limitations to bending down and kneeling (item 11), and driving requires the need to change position (item 2). Driving and work were also activities that may not be engaged in daily by all individuals and therefore not appropriate as an item within a measure of everyday functional impact.

During the cognitive debriefing, overall feedback on the RMDQ was positive and supported the content validity of the measure. Details of participants’ feedback are provided in Table 4. All participants reported that it was comprehensive and easy to understand and complete.

**Table 4:** Item relevance and participants’ suggested edits

RMDQ - item number and summary	Relevant^a^ Yes (n/N)	Understanding/ Comprehension^b^ Yes (n/N)	Participant quote on Understanding/Comprehension^c^	Participant suggested change Yes (n/N)^d^	Participants suggested change and quote
1 – I stay at home most of the time because of my back	Y (22/23)	Y (23/23)	“Does it (CLBP) limit me from going out and doing the things that I need to do, running errands, going out to … um, for entertainment purposes or just anything” P01–002	Y (1/23)	“Mine hasn’t gotten so bad that it’s made me do anything like that, so it seems like an irrelevant question to me [so could be removed]” P02–008
2 – I change position frequently	Y (23/23)	Y (23/23)	“do I need to sit with proper posture, or put weight on one side or the other … in order to be able to sit and be comfortable when I’m sitting” P01–003	N	Not applicable
3 – I walk more slowly	Y (23/23)	Y (23/23)	“I was recalling to try to see if I could remember myself having to walk slow due to my back pain” P01–008	N	Not applicable
4 – Not doing jobs around home	Y (23/23)	Y (23/23)	“Not being able to lift heavy objects around the house. Again, take the trash out” P01–008	Y (3/23)	“It’s uh, I’m not doing any, the word any, you know, I have a very wide range of jobs I have to do around the house you know, so that’s a little confusing to me. You know if it’s, does that mean any of the things that I do or is it just the more physical things” P02–001 “cause of my back, I’m not doing any of the jobs that I usually do around the house. I put no, but there’s some things that I don’t do around the house because of my back” P02–008 “That was one I couldn’t answer. Because it depends. Some days I can like I said, when I mop the floor … it puts me in a very bad position because it makes my back hurt more. When I do that I’m done and there are some days I can’t do it at all” P01–002
5 – I use a handrail to get upstairs	Y (23/23)	Y (23/23)	“it’s asking me if my back pain severe, so severe that I need assistance getting up the stairs or walking assistance” P01–008	Y (2/23)	[Should it be reworded?] “Hm, yes. Probably like because of my back, I need assistance getting upstairs. Sometimes, some stairs don’t even have handrails, you know? P01–008 “It was a little not confusing, but like some of these are, like especially the first one [points to item 5], it isn’t really relevant for just asking how are you doing today.” also suggested “Maybe you know [for item 5 & 6] there would be like a box, you know, often, frequently, you know, like 1–2 times a week, you know what I mean?” P02–003
6 – I lie down to rest	Y (23/23)	Y (23/23)	“Like I lay down to rest more often.” P03–005	Y (3/23)	“Because in a week’s time, you’d have some good days and bad days, so you can average them out …. It’s like, well, number 6, “Because of my back, I lay down to rest more often.” Uh, that’s, that’s just say-, you know, to me, that’s, it depends if it, if you’re, if it hurts real bad, you know, you maybe, may lie, lay down for a while.” P02–008 “You know? Um, I don’t know. And because of my back, I lay down to rest more often. That seems like that general, broad question... it’s like, you know, is that really have anything to do with today?” P02–003 “Um, “Because of my back, I lie down to rest more often.” Um, on occasions I will, I will lay down but not, it’s not an often type situation.” P03–003
7 – Hold on to get out of an easy chair	Y (23/23)	Y (23/23)	“The way I get out of a chair. (laughs) I mean I’m not, I’m not using my upper body to … I may lean forward more” P01–001	Y (1/23)	“Uh, number seven, an easy chair, what, how would you describe an easy chair?... maybe, like you said a recliner, you guys could add that in there.” P01–006
8 – I try and get other people to do things for me	Y (23/23)	Y (23/23)	“Um, yes for that. Occasional if there’s a, if there’s heavy lifting, or um, or uh, constantly moving from point A to point B with a heavy object, or something I previously would have, um, been able to tackle myself, then, um, I know, I know my limitations because of back. Um, I’ll get some help.” P01–003	Y (3/23)	“Uh, 8, 8 is a little strange for me. I’m not sure, uh, other people are … I, I don’t know. Just it, it sounds awkward to me … Maybe like other people offer to help me because they see that I’m not as agile as I used to be” P01–001 “[should be reworded] “Um, I would say, due to my back, I often, sometimes need assistance form people.” P01–008 “That’s a general, that’s a general thing too. Um, that would be another good one to say, you know, every day or frequently or just today, um, or instead of “I try,” [maybe reword to] “I tried to get other people to do things for me today”. Maybe for, you know, so” P02–003
9 – I get dressed more slowly	Y (22/23)	Y (23/23)	“I answered it no. Because I don’t feel like um, I, I dress any slower, I just do it more carefully.” P02–007	Y (2/23)	“The only thing I would see is because where it says, bec- … I get dressed more slowly than usual because of my back, I would put back pain. That would be it”. P01–002 “I just feel like that question (item 9) does not fit in whatsoever in the questionnaire” P01–008
10 – I only stand for short periods	Y (23/23)	Y (23/23)	[I was thinking] “Because I can’t stand be, been, uh, stand up for a long period, so I, I do different kind of movements, like sitting down, um, stand up, because it, it bother” P01–007	N	Not applicable
11 – I try not to bend or kneel	Y (23/23)	Y (23/23)	[I was thinking] “Like if I have to pick up something, say if I drop something or I have to pick up something, depending on what it is, I’m like, “Oh Lord, I got it,” you know, but I have to do what I have to do.” P01–002	N	Not applicable
12 – I find it difficult to get out of a chair	Y (23/23)	Y (23/23)	[I was thinking] “Uh, a chair with or without arms, a plastic chair, a wooden chair, a chair that has a pad on it. Any of those type of chairs. I don’t, I don’t really have a problem getting up.” P01–003	N	Not applicable
13 – Back is painful most of the time	Y (23/23)	Y (23/23)	[it’s asking] “Do you feel pain more than you don’t feel pain.” 03–002	N	Not applicable
14 – Turn over in bed	Y (23/23)	Y (23/23)	“I was thinking that sometimes, I have to turn round a lot in bed … Like toss and turn. I have to accommodate myself by putting a pillow between my legs sometimes and I only sleep comfortably on my left side, as far as I’ve been noticing.” P01–008	N	Not applicable
15 – Appetite is not very good	Y (21/23)	Y (23/23)	“I was, um, thinking about well my back hurts tonight I’m not gonna eat. (laughs) You know if I feel like eating I will, if I don’t, I won’t..” P01–002	Y (2/23)	“not really [relevant]. Only because I’ve never had an issue with appetite because of back pain” P02–003. “The appetite thing was weird [not sure if it was needed]”.02–005
16 - Putting on socks (or stockings)	Y (23/23)	Y (23/23)	“Um, yes, on occasion. And I, I’m kind of picturing myself, you know, bending over, sitting on the bed, and trying to do it. Or just crouching down on the floor, trying to tie my shoes and, and there is some pain there” P01–003	Y (2/23)	“Um, I think again, if it’s just thinking of today, I would put, “Today, I had trouble putting on my socks.” P02–003 “um, you really should put like every, like I said “bend your knee,” or like, if you’re standing up and you’re just, hold on to something and put on your sock.” P01–006
17 – Walk short distances	Y (23/23)	Y (23/23)	[I was thinking] “you know, like in, in my position I cannot just, I cannot walk, walk for a long period, so I was thinking of myself.” P01–007	Y (1/23)	“It’s like, “Today, I only walked a short distance because of my back,” [inclusion of today] P02–003
18 – Sleep less well	Y (23/23)	Y (23/23)	“I was thinking about exactly what it says, how do I sleep.” P01–002	Y (2/23)	“I would say … Ah, how I would word this? My quality of sleep is not as good … is not as good … is not what it should be because of my back pain.” P01–002 “I’ll probably say, do you have trouble sleeping at night due to back pain?” P01–008
19 – I get dressed with help	Y (23/23)	Y (23/23)	“[it’s asking] Does anybody else put on clothes for me?” P01–006	N	Not applicable
20 – I sit down most of the day	Y (23/23)	Y (23/23)	“I said, uh, yes, I do sit down because of my back. That’s for sure, and I also stand up too.’ P01–005	Y (1/23)	“I sit down for the, for the most of the day because of my back. Yes, that’s a relevant question but there is some that probably you’d have to ask from a different time period. Like” P01–008
21 – I avoid heavy jobs	Y (23/23)	Y (23/23)	“No. Just avoiding heavy jobs, period.” P03–007	Y (1/23)	“I would say, “Today, I avoided a heavy job because of my back” [inclusion of today] P02–003
22 – Irritable and bad tempered with people	Y (23/23)	Y (23/23)	[I was thinking] “I’m … It’s like I’m upset with myself and it carries over to other people” P01–001	Y (3/23)	“Um, again, I would just put like partial or sometimes … you know, it might not apply to someone everyday but it does apply to them sometime throughout the course of their day or, you know” P03–003 **“**so, using today, I said no, but I mean, in general, yeah, I think, you know, if you’re having more increased pain, you’re going to be a little cranky crank. (laughs). You know?” P02–003 “The yes or no doesn’t really [make sense]... For me, it doesn’t because, you know, that sort of says, because of my back, I’m more irritable. That makes me think you’re irritable all the time … [but it] depends on the severity of the pain.” P02–008
23 – I go upstairs more slowly	Y (23/23)	Y (23/23)	[I was thinking] “Yes, I wouldn’t go up, running stairs” P01–006	Y (1/23)	“I didn’t answer number 23 because it’s … it’s because of my back I go upstairs more slowly than usual and like I said I avoid stairs, period.” P01–002
24 – I stay in bed most of the time	Y (23/23)	Y (23/23)	“Um, I guess 24 is basically saying, you know, um, I stay in bed all day long because of my back pain.” P03–003	Y (1/23)	“I stay in bed most of the time because of my back.” Uh, that would be like on a day when it’s really, really bad … Some people may, but it just seems more of a broad question, rather than just for today.” P02–008

^a^This column highlights if any participants raised concerns over the relevance of items

^b^This column represents if any participants raised concerns with the comprehension or understanding of items

^c^This column presents a participant quote following being asked “What were you thinking about?”

^d^This column represents whether participants have suggested amendments to this item and displays the number of participants who made suggestions

When participants discussed items that referred to activities being done ‘more slowly’ [item 3 walking slowly; item 9 dressed more slowly; item 23 go upstairs more slowly] participants not only reported thinking about how they do things more slowly with CLBP compared to before having CLBP, they also spoke about how, on days when their CLBP was worse than usual, they would undertake these activities at a slower pace, meaning they would take longer to complete a task than usual to try to avoid worsening their pain even further.

A few participants had comments, queries, or suggestions for improvements to the RMDQ instructions (*n* = 4) and recall period (*n* = 3), but these were not consistent across participants; and others gave positive general feedback on these elements of the measure but raised minor suggestions for improvement during the debriefing (recall period *n* = 5 and response options *n* = 8). However, these were not consistent and, upon review, felt to be likely due to the unusual circumstances of one-off completion of the measure during the interview rather than an issue with the measure itself. For example, querying the recall period of “today” as it applied to current one-off use in the interview would not be relevant to the measure as administered in a clinical trial.

Feedback on individual items within the RMDQ suggested that all items were relevant to almost all participants. Only three items were queried for relevance by four participants (two queried one item, the other two items were each queried by one participant). Specific feedback can be seen in Table 4. One participant queried item 1 (staying at home) because their own back pain had never been so severe (they had moderate CLBP); one other participant with moderate CLBP felt that item 9 (getting dressed more slowly the usual) did not fit in with the questionnaire; and two participants (one mild, one moderate CLBP) felt that item 15 (appetite is not very good) was not relevant to them. However, other participants did discuss how they would sometimes not feel like eating because they were in so much pain “*I was, um, thinking about well my back hurts tonight I’m not gonna eat” P*01–002. Additionally, although they reported the items to be relevant to them, nine participants made suggestions for minor wording edits to several items. However, the suggestions reflected preferences and were not reported across the sample.

Overall there was no consistent feedback that suggested changes were necessary to support the content validity of the tool. Improvements made to the measure in terms of the addition of dichotomous response options were well received and clearly understood. Participants were not explicitly asked to comment on the format of the RMDQ, since screenshots of the electronic tablet were being used, however, no participant raised any concerns with the style and format of the RMDQ during the interview.

## Discussion

Qualitative data from individuals with CLBP highlighted the substantial burden of CLBP and its impact on participants lives. The results confirmed that the RMDQ has content validity. Although the common use of the RMDQ may suggest acceptance within the clinical community of content validity, it is an evidentiary expectation, and this should be shown qualitatively through direct feedback from patients. The conceptual model, developed using the qualitative data from this study, illustrates the symptoms and everyday functional impacts associated with CLBP that are salient to those with this condition. This demonstrates the burden of CLBP on both the individual and society, which is supportive of existing research [2–5].

When the key symptoms and functional impacts highlighted in the conceptual model were mapped to the RMDQ, this highlighted that the RMDQ was comprehensive and all symptoms and impacts identified as important by participants were captured. Although three of the concepts (washing, driving, and work) identified in the conceptual model were not explicitly captured in the RMDQ, upon review of participant quotes it was felt these were either: adequately encapsulated within other RMDQ items (ie, washing and driving); were concepts that not everyone would take part in (ie, driving, work); or were activities that could be better captured using other more specific measures (ie, work). Therefore, it was felt that these were not appropriate to include as specific items within the RMDQ, which is a measure of everyday functional impact.

Two of the concepts captured in the RMDQ (lying down and appetite) were identified by almost all participants as being important during the CD section of the interview (when prompted by the RMDQ item) however they were not raised spontaneously during CE and so the discussion around these was not as in-depth as other concepts. Although it was felt that the feedback from patients supported the importance and relevance of these concepts, it would be interesting to explore these in more detail to clarify their impact in CLBP.

Minor changes that had been made by the authors to the RMDQ for this study (changes to the layout for electronic administration and the addition of explicit dichotomous response options) were well received by participants and added clarity to their responses, allowing a distinction to be drawn between missing and “no” responses. Participants did not raise any concerns with the RMDQ being presented to them as screenshots from an electronic tablet format, where any changes to format and layout from the paper version could clearly be seen. Feedback on the usability of the electronic devices has been evaluated elsewhere [28].

All participants reported that the RMDQ was easy to understand and complete. The RMDQ was considered comprehensive and relevant, capturing all concepts relevant to participants. Although a few participants had minor comments, queries, or suggestions for improvement (for example to wording, items, recall period, or response options), there was no consistent feedback across participants and no changes to the measure were considered necessary within the proposed context of use.

Despite the RMDQ being developed prior to FDA Guidance for Industry on Patient-Reported Outcomes [25], the findings consistently demonstrate that the RMDQ has content validity. This is an essential step in the development of PRO measures [25]. When taken into consideration alongside the results of previous studies evidencing good psychometric properties [14, 17–24, 32], the current study complements this to indicate that the RMDQ is a reliable and valid (including responsive) measure of every day physical functioning in CLBP, suitable for its proposed context of use.

The number of participants involved in this study reflects the typical sample size for in-depth qualitative research. Although participants were all recruited from the US, multiple locations were used to maximize geographical diversity, and although there was a spread of age, gender, education level, pain severity and time since diagnosis, the sample population is predominantly a White and Black/African American non-Hispanic population; other ethnic groups, such as Asians, are not represented. Therefore, it would be beneficial to confirm the cultural representativeness of these findings. Saturation was reached with no new concepts being discussed in the last batch of interviews. It was not feasible to use the electronic tablet-based administration during the interviews, so screenshots of the RMDQ from the electronic device were presented to the participant for pen and paper completion. However, the usability and feasibility of this electronic mode of administration have been evaluated in a separate study [28].

## Conclusions

The RMDQ was intended to capture the everyday functional impact of CLBP, which by the nature of the condition, is primarily centered on physical function. This is, therefore, a core aspect of any clinical outcome assessment measurement strategy in the evaluation of CLBP. The findings from the current qualitative research demonstrate that the RMDQ has content validity, reflects concepts experienced by participants with CLBP, and that the changes made to the measure for electronic completion were understood and suitable.

## Data Availability

The datasets generated and/or analyzed during the current study are not publicly available due to the sensitive nature of the questions asked in this study but are available from the corresponding author on reasonable request.

## Abbreviations

CLBP: Chronic low back pain
CD: Cognitive debriefing
CE: Concept elicitation
FDA: Food and Drug Administration
HRQoL: Health-related quality of life
PRO: Patient reported outcome
RMDQ: Roland Morris Disability Questionnaire
US: United States
